# Genome-wide haplotype association study identify TNFRSF1A, CASP7, LRP1B, CDH1 and TG genes associated with Alzheimer's disease in Caribbean Hispanic individuals

**DOI:** 10.18632/oncotarget.6391

**Published:** 2015-11-25

**Authors:** Zhenwei Shang, Hongchao Lv, Mingming Zhang, Lian Duan, Situo Wang, Jin Li, Guiyou Liu, Zhang Ruijie, Yongshuai Jiang

**Affiliations:** ^1^ College of Bioinformatics Science and Technology, Harbin Medical University, Harbin, China; ^2^ Genetic Data Analysis Group, The Genome Science Consortium, Harbin, China; ^3^ Genome Analysis Laboratory, Tianjin Institute of Industrial Biotechnology, Chinese Academy of Sciences, Tianjin, China

**Keywords:** Alzheimer's disease, haplotype association analysis, Pathology Section

## Abstract

Alzheimer's disease (AD) is an acquired disorder of cognitive and behavioral impairment. It is considered to be caused by variety of factors, such as age, environment and genetic factors. In order to identify the genetic affect factors of AD, we carried out a bioinformatic approach which combined genome-wide haplotype-based association study with gene prioritization. The raw SNP genotypes data was downloaded from GEO database (GSE33528). It contains 615 AD patients and 560 controls of Caribbean Hispanic individuals. Firstly, we identified the linkage disequilibrium (LD) haplotype blocks and performed genome-wide haplotype association study to screen significant haplotypes that were associated with AD. Then we mapped these significant haplotypes to genes and obtained candidate genes set for AD. At last, we prioritized AD candidate genes based on their similarity with 36 known AD genes, so as to identify AD related genes. The results showed that 141 haplotypes on 134 LD blocks were significantly associated with AD (P<1E-4), and these significant haplotypes were mapped to 132 AD candidate genes. After prioritizing these candidate genes, we found seven AD related genes: APOE, APOC1, TNFRSF1A, LRP1B, CDH1, TG and CASP7. Among these genes, APOE and APOC1 are known AD risk genes. For the other five genes TNFRSF1A, CDH1, CASP7, LRP1B and TG, this is the first genetic association study which showed the significant association between these five genes and AD susceptibility in Caribbean Hispanic individuals. We believe that our findings can provide a new perspective to understand the genetic affect factors of AD.

## INTRODUCTION

Alzheimer's disease (AD) is an acquired and incurable disorder of cognitive and behavioral impairment with a long and progressive course [1]. It's the most common form of dementia. Currently, AD affects over 35 million people worldwide, and the incidence is apparently rising year by year [2]. The etiology of AD is complex. A variety of factors contribute to the chance of developing AD, such as ageing, immune dysregulation, inflammatory damage, genetic and environmental factors [3, 4]. AD can be classified into two forms as early-onset and late-onset [5]. The early-onset AD is caused by rare autosomal dominant mutations, and it generally appears before age 65. However the late-onset form appears after age 65. The late-onset form is modulated by genetic variants with relatively low penetrance, but high prevalence, it accounts for more than 95 percent of all cases of AD [6]. Thus age is a principal risk factor for AD, as more people are living longer, the number of people with AD is expected to increase significantly in future. So it is necessary to find out the pathogenic mechanism of AD.

Some studies have shown that genetic variants play an important role in the development of AD. Recently, numerous genome-wide association studies have been published for AD, and many AD susceptibility loci have been identified, such as APOE, LRAT, APOC1, SORL1, GAB2, PGBD1, CHRNB2, CLU, PICALM, CR1, PCDH11X and TRPC4AP [7-17]. Besides, variants in some AD related genes have been replicated in several independent cohorts and were investigated the association with AD by meta-analysis [18, 19]. In addition, Bertram L et al. even built up a database that comprehensively cataloged all genetic association studies in the field of AD and did systematic meta-analysis on these studies [20]. Although many AD susceptibility genes have been identified, they can only explain a portion of the pathogenesis of AD. In order to further explore the pathogenesis of AD, we attempt to identify novel genes associated with AD.

As we know, Linkage Disequilibrium (LD) exists among SNPs. Haplotype is made up of tightly linked SNPs that are on a chromosome or a region, so it contains genetic information of several SNPs. Previous studies have confirmed that in the related analysis between loci of LD and complex diseases, association analysis based on haplotype can achieve better statistical analysis results than on single SNP, and it is more conducive for identifying disease genes [21-23]. Moreover, some researchers have found that functionally related genes generally share some common characteristics, such as gene expression pattern and gene regulation pathways, and these genes often lead to similar phenotypes [24, 25]. So based on the hypothesis of genotype–phenotype associations, we can prioritize disease candidate genes based on their similarity with known disease genes to discover potential disease associated genes [25, 26]. In the current study, we integrated haplotype association analysis with gene prioritization to mine AD related genes.

## RESULTS

### The results of genome-wide haplotype association study

In this study, we carried out a genome-wide haplotype association study using SNP genotypes data of 1,175 Caribbean Hispanic individuals (GSE33528). There are 628,670 SNPs on 22 autosomal chromosome in raw data set passed the quality control (MAF >1E-3, H-W P > 1E-3 and call ratio >75%). Then by the FGT method, we identified 131,790 LD blocks and 615,361 haplotypes. For each haplotype, we carried out a chi-square test to obtain its statistical significance. At last, we found 141 haplotypes that were significantly associated with AD (*P* < 1E-4, see Supplementary Table 2 in the website: http://www.bioapp.org/research/ADhaplotype). The 141 significant haplotypes were located on 134 block regions.

### Mapping and prioritizing the AD candidate genes

We mapped the 141 significant haplotypes to genes based on their physical location information on chromosomes, and obtained 132 AD candidate genes (see Supplementary Table 3 for more detailshttp://www.bioapp.org/research/ADhaplotype). Then we prioritized these AD candidate genes according the features from 19 data sources. For each data source, we got a prioritized list of the candidate genes. On the bases of this, we finally obtained a global rank for each candidate genes by P-value which represented the degree of similarity with known AD genes. The detailed results of candidate genes prioritization were shown in the Supplementary Table 4 (http://www.bioapp.org/research/ADhaplotype). According to the final prioritization result, the top seven genes on the rank list are significantly similar with known AD genes (*P*-value <0.001), they are APOE, APOC1, TNFRSF1A, LRP1B, CDH1, TG and CASP7 (listed in Table 1).

**Table 1:** The top seven AD related genes of prioritization

Rank	Ensembl gene ID	Gene symbol	*P*-value
1	ENSG00000130203	APOE	1.21E-09
2	ENSG00000130208	APOC1	1.09 E-06
3	ENSG00000067182	TNFRSF1A	3.63E-04
4	ENSG00000168702	LRP1B	3.84 E-04
5	ENSG00000039068	CDH1	4.88 E-04
6	ENSG00000042832	TG	6.21 E-04
7	ENSG00000165806	CASP7	9.5 E-04

We compared the candidate genes with known AD genes, the candidate genes were ranked based on P-values of global prioritization. The result of the top seven candidate genes is listed in the Table 1. Their *P*-values is lower than 0.001.

### The analysis of gene APOE and APOC1

**Figure 1:**
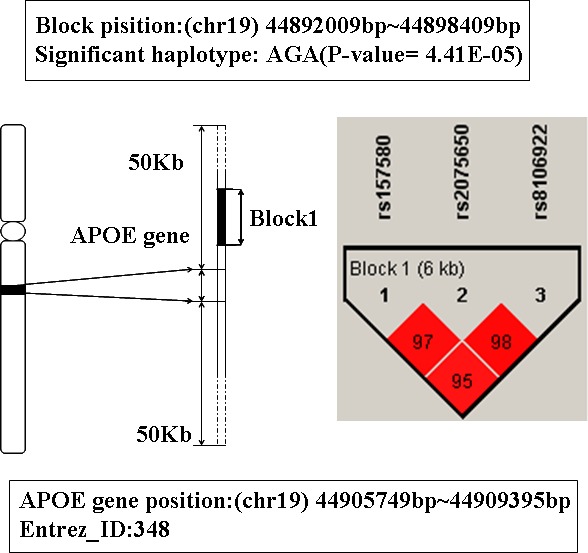
The haplotype analysis result of APOE gene

**Figure 2:**
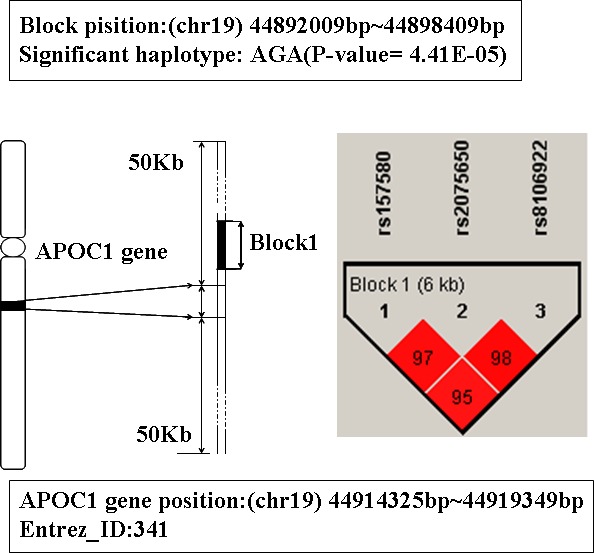
The haplotype analysis result of APOC1 gene

In Table 1, the top two genes are APOE and APOC1, they are located on 19q13.2. These two genes were mapped by the same LD blocks which composed of 3 SNPs. The detail is shown in Figure 1 and Figure 2. The haplotype AGA in this block is significantly associated with AD, and its *P*-value of Chi-square test is 4.41E-05. The other haplotypes of this block are AAG, AAA and GAA. It's noteworthy that APOE and APOC1 are known AD susceptibility genes in OMIM database (OMIM ID: 602710). They are also the only genes that exist both in candidate genes set and training set.

### The analysis of gene TNFRSF1A

**Figure 3:**
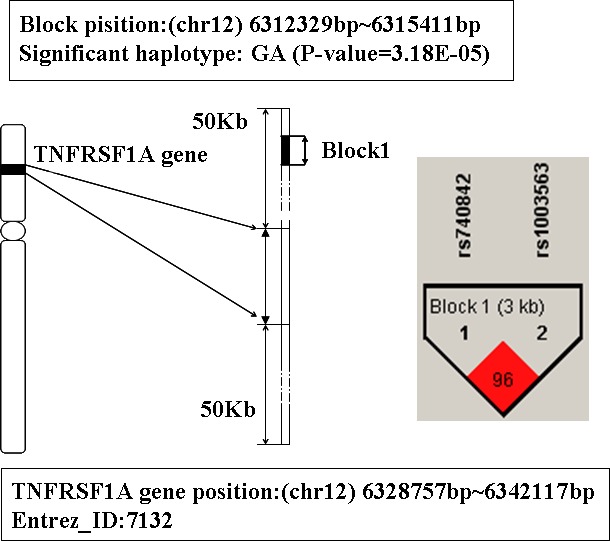
The haplotype analysis result of TNFRSF1A gene

The gene TNFRSF1A is ranked third in Table 1. This gene is located on chromosome 12p13.2, its physical location is from 6328757bp to 6342117bp. The detailed information about this gene is shown in Figure 3. There is a LD block which consists of 2 SNPs (rs740842 and rs1003563) mapped to this gene. And there are 3 hapotypes AG, GA and GG on this block. Among them, the haplotype GA showed significant association with AD (*P*-value is 3.18E-5), and people with this haplotype may decrease the risk of AD susceptibility.

The protein encoded by TNFRSF1A is one of the major receptors for tumor necrosis factor –alpha (TNF-alpha). TNF-alpha is the significant family members of TNF which is extremely important signaling protein in the immune system [27]. TNFRSF1A can activate NF-kappaB, mediate apoptosis, and its function as a regulator of inflammation [28]. Some previous studies indicated that inflammatory mechanisms have been implicated in a series of neuropsychiatric conditions including behavioral disturbances, cognitive dysfunction, and affective disorders [29, 30]. AD is a neurodegenerative disorder, characterized by chronic and disabling memory impairment, cognitive dysfunction, language barrier, and there are frequently changes in behaviour and personality [31]. Inflammatory events are implicated in AD [32, 33]. Additionally, in OMIM database TNFRSF1A is reported susceptible to multiple slerosis (MS) (OMIM ID: 191190). MS is also an inflammatory and nervous system disease as AD [34]. From the above, we infer that TNFRSF1A maybe an AD related gene. To our knowledge, this is the first genetic association study which shows the significant association between TNFRSF1A and AD.

### The analysis of gene LRP1B

**Figure 4:**
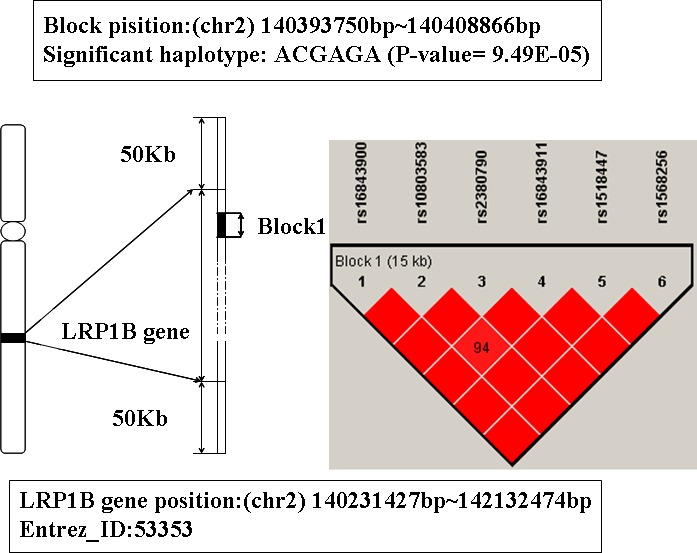
The haplotype analysis result of LRP1B gene

In Table 1, the gene behind TNFRSF1A is LRP1B. It is located on chromosome 2q21.2. The haplotype block that mapped to this gene includes 6 SNPs: rs16843900, rs10803583, rs2380790, rs16843911, rs1518447, and rs1568256 (showed in Figure 4). The haplotype ACGAGA showed significant association with AD (*P*-value is 9.49E-5). The individual who carries this haplotype may increase the susceptibility of AD.

LRP1B belongs to the low density lipoprotein (LDL) receptor gene family. These receptors play a wide variety of roles in normal cell function [35]. A study of genome screen have identifies that LRP1B is significant and protective for successful aging without cognitive decline [36]. While, AD often starts in senium or presenium and one of the significant characteristic of AD is cognitive dysfunction [2]. In addition, EST database analysis indicates that LRP1B is expressed in skeletal muscle and thyroid gland, as well as in brain lesions of patients with MS [37]. Also Liu et al. have detected LRP1B expression in all regions of the adult brain examined and thyroid gland by using RNA dot blot analysis [35], and more and more evidence reveal the relationship between the thyroid function and the pathogenesis of AD [38]. So we infer that LRP1B may be associated with AD.

### The analysis of gene CDH1

**Figure 5:**
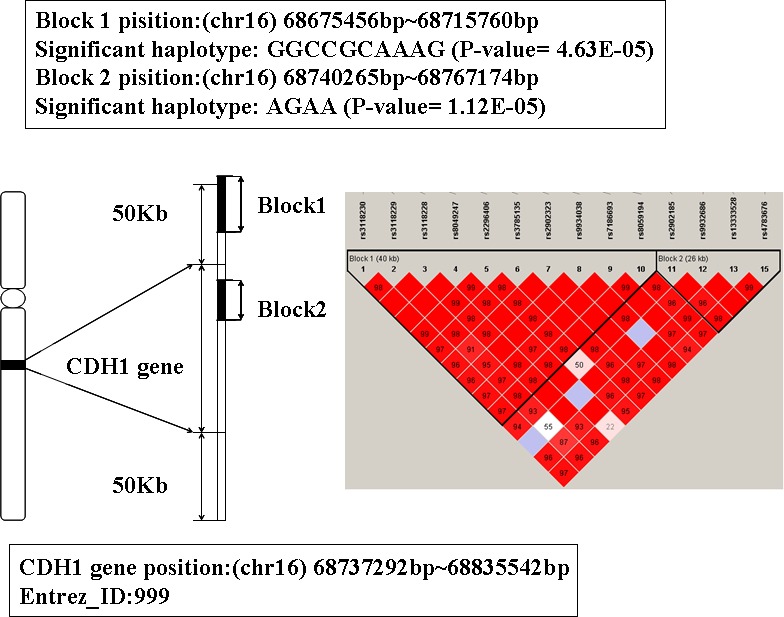
The haplotype analysis result of CDH1 gene

The location of CDH1 is 16q22.1 from 68737292bp to 68835542bp. There are two significant haplotypes mapped to this gene (the P-value are 4.63E-05 and 1.12E-05 respectively). One LD block is consisted of 10 SNPs and the other one includes 4 (showed in Figure 5). The significant haplotype of the first block is GGCCGCAAAG. The second block has 5 haplotypes and the significant haplotype is AGAA. Individuals have these haplotypes may decrease the risk of AD susceptibility.

CDH1 is a co-activator subunit of anaphase-promoting complex/cyclosome (APC/C), and it is important for the regulation of cell cycle. Mutations in this gene are correlated with gastric, breast, colorectal, thyroid and ovarian cancer (OMIM ID: 192090). In addition, studies have shown that CDH1 was expressed highly in the mammalian neurons, and it has the function of regulating synaptic differentiation and transmission [39, 40]. CDH1 plays an important role in the development of central nervous system disease [40, 41], we deduce the gene CDH1 is correlated with AD.

### The analysis of gene TG

**Figure 6:**
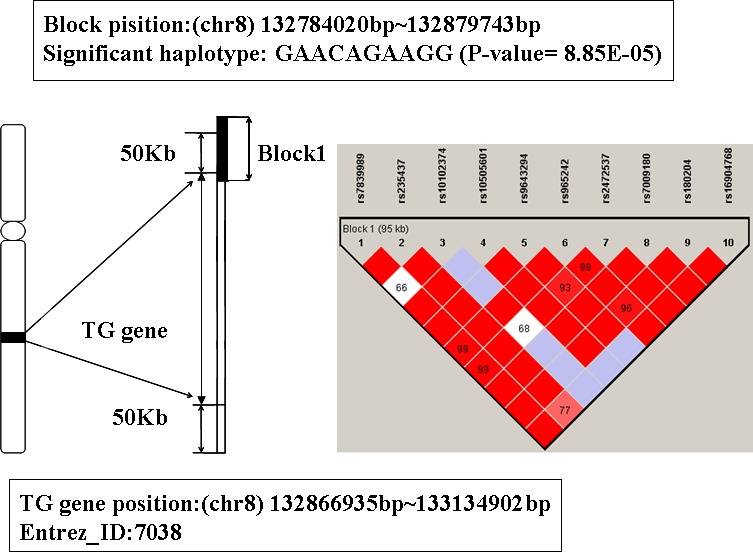
The haplotype analysis result of TG gene

The global prioritization P-value of gene TG is 6.21 E-04 (showed in Table 1). Its physical location is from 132866935bp to 133134902bp on chromosome 8. The LD block contains 10 SNPs mapped to TG, see Figure 6 for detailed information. The significant haplotype of this block is GAACAGAAGG (P-value is 8.85E-5). People with this haplotype may decrease the risk of AD susceptibility.

Thyroglobulin (TG) is a glycoprotein homodimer produced predominantly by the thryroid gland. Mutations in this gene cause thyroid dyshormonogenesis, and are associated with moderation to severe congenital hypothyroidism [42, 43]. Polymorphisms in this gene are associated with susceptibility to autoimmune thyroid diseases (AITD) such as graves disease and hashimoto thryoiditis [44]. Some studies have indicated that there was some relationship between thyrotropin level and AD risk [45]. Thyroid disease has been researched as a risk factor for AD, and the history of thyroid function decline may increase the relative risk of suffering from AD [46, 47]. Thus, we infer TG may be a susceptibility gene for AD.

### The analysis of gene CASP7

**Figure 7:**
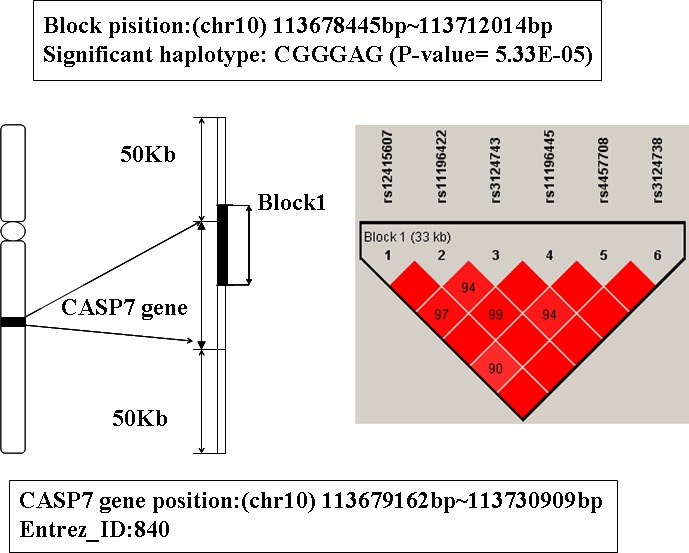
The haplotype analysis result of CASP7 gene

The global prioritization P-value of gene CASP7 is 9.5 E-04. Its location is 10q25, from 113679162bp to 113730909bp. A LD block including 6 SNPs was mapped to this gene (see Figure 7). There are 7 haplotypes on this block, among them, the haplotype CGGGAG shows significant association with AD (P-value is 5.33E-5). People with this haplotype may decrease the risk of AD susceptibility.

CASP7 encodes a member of the cysteine-aspartic acid protease (caspase) family. Studies have shown that sequential activation of caspases plays a central role in the execution-phase of cell apoptosis, and CASP7 is also involved in inflammation [48, 49]. Moreover, previous studies have found CASP7 is activated in microglia in the ventral mesencephalon of Parkinson's disease (PD) and the frontal cortex of individuals with AD [50]. Microglia is necessary for normal brain function. However, uncontrolled and over-activated microglia can trigger neurotoxicity, and are a prominent source of pro-inflammatory factors [51]. Research indicated that CASP7 is involved in regulating microglia activation, and knockdown or chemical inhibition of it hindered microglia activation and consequently reduced neurotoxicity, while activation of microglia and inflammation-mediated neurotoxicity are speculated to play a decisive role in the pathogenesis of several neurodegenerative disorders such as AD, MS, and PD [50, 52]. So we infer that CASP7 may be an associated gene of AD.

For all of the above seven genes, the P-values of global similarity was less than 0.001, and at least one haplotype in or nearby the gene region was significantly associated with AD (*P*<1E-4). We have also found that these genes indeed had some relationship with AD from previous researches, so we inferred they were AD related genes.

## DISCUSSION

Genetic factors play an important role in the development of AD, however the identification of genetic factors related to AD is still a challenge. Here, we combined genome-wide haplotype association study with gene prioritization to mine AD related genes. The results showed that this strategy could overcome the limitation of traditional analysis methods and identify genes associated with AD effectively. In our study, we identified seven AD related genes: APOE, APOC1, TNFRSF1A, LRP1B, CDH1, TG and CASP7.

APOE and APOC1 that ranked top two on the priority list are known AD genes in OMIM database. In consideration of they are the only genes exit both in training set and candidate genes set, we eliminated these two genes from training set, and performed the prioritization again on 132 AD candidate genes. The result is shown in Table 2. APOE is still the most significant gene which is similar to known AD genes. APOC1 is also ranked forward in the result list of prioritization, and its global P value of prioritization is 1.49E-05. This result can illustrate the robustness of prioritization, and through prioritization we can obtain highly associated genes of AD from candidate genes reliably.

**Table 2:** The result of prioritization for reliability verification

Rank	Ensembl gene ID	Gene symbol	*P*-value
1	ENSG00000130203	APOE	1.49E-05
2	ENSG00000168702	LRP1B	5.41E-05
3	ENSG00000067182	TNFRSF1A	2.09 E-04
4	ENSG00000039068	CDH1	4.4 E-04
5	ENSG00000042832	TG	4.84 E-04
6	ENSG00000184845	DRD1	8.55 E-04
7	ENSG00000165806	CASP7	1.28 E-03
8	ENSG00000130208	APOC1	3.63 E-03
9	ENSG00000101197	BIRC7	4.46 E-03
10	ENSG00000142515	KLK3	7.42 E-03

In order to verify the reliability of prioritization, we eliminated the two known AD genes APOE and APOC1 from training set, and performed the prioritization again on 132 AD candidate genes. The result of the top ten candidate genes is listed in the Table 2.

Among the seven genes, except gene APOE and APOC1, the other five are novel genes that reported to be associated with AD. The global P-values of these genes indicated that they had higher similarity with known AD genes. Through reviewing related literatures, we found that all these genes had some relationship with AD. The protein which is coded by gene TNFRSF1A can mediate apoptosis and inflammation [32, 53]. CDH1 is important to the development of central nervous system disease. Gene CASP7 is also involved in inflammation, and it regulates the activation of microglia which is necessary for normal brain function. Besides, gene LRP1B is significant for successful aging without cognitive decline. Gene TG is associated with thyroid diseases, while the level of thyrotropin has some relationship with the risk of developing AD. AD is a common and important neurodegenerative disorder, its pathogenesis includes strong interactions with immunological mechanisms in the brain [54, 55]. Aging, inflammatory damage, and thyroid disease have been considered as affect factor of developing AD. According to our analysis TNFRSF1A, CHD1, CASP7, LRP1B and TG might be important associated genes of AD. These indicate that our method is effective and the results are reliable. We believe that our results can provide a new perspective to understand the genetic affect factors of AD.

## MATERIALS AND METHODS

### Datasets

In our study, the raw SNP genotypes data was downloaded from GEO database (http://www.ncbi.nlm.nih.gov/geo), the GEO accession is GSE33528. It was released on May 01, 2012. The purpose of original study was to survey large rare copy number variations in late-onset AD, and they had found some meaningful results [56]. The datasets include 615 patients with late-onset AD and 560 controls of unrelated Caribbean Hispanic individuals. Its SNP chip platform is GPL14932 which includes 662,841 SNP markers. Obviously, the sample size of this AD case-control datasets is big enough and it is very precious, so we expect to reuse it to mine novel AD related genes.

### Genome-wide haplotype association analysis

Firstly, we applied quality control to filter the 643,353 autosomal SNPs of raw data. The criteria of quality control were as follows: (1) Minor allele frequency (MAF) > 0.001; (2) Hardy–Weinberg equilibrium (H-W) test P > 1E-3; (3) Percentage of individuals successfully genotyped >75%. The SNPs that passed the quality control were used for the subsequent haplotype association analysis.

Here we used the software Haploview to identify the LD blocks and performed association analysis. The method of identify LD blocks was Four Gamete Tests (FGT) which combined the impact of genetic factors into block construction. Then the Maximum Likelihood Estimation and Expectation Maximization algorithm were used to estimate the haplotypes and their frequencies. At last, the statistically significant haplotypes that associated with AD were obtained by chi-square test [57, 58]. A haplotype was considered associated with AD, if its P value of chi-square test was less than 1.0E-4.

### Mapping AD candidate genes

The AD related haplotypes were mapped to genes based on the location information of LD blocks and human genes. Physical location information of human genes was obtained by querying the NCBI gene database (ftp://ftp.ncbi.nlm.nih.gov/genomes/H_sapiens/mapview/seq_gene.md.gz). If a gene located within 50 kb upstream or downstream of AD related haplotypes [59], it was regard as a candidate gene that might be related to AD. By means of this, we obtained the candidate genes set of AD.

### Prioritizing candidate genes to find AD related genes

Many researches have indicated that genes related to the same disease often similar in some aspects, such as expression, functional annotations, and regulation information [60, 61]. So in our research, we hypothesized that genes associated with AD shared some genetic characteristics. Therefore, we can identify the potential AD related genes from AD candidate genes based on their similarity with known AD genes.

First, we got the training set of AD genes. The gene that had been verified having strong association with AD by more than three published studies was regarded as a known AD gene. We had collected 36 AD genes as training genes (see Supplementary Table 1 for more details in the website: http://www.bioapp.org/research/ADhaplotype) through querying Genetic Association Database (GAD) and the Online Mendelian Inheritance in Man (OMIM) database. Second, feature data of AD is gathered based on training genes by consulting various data sources. Here, 19 data sources were used, these data sources can be divided into seven categories: ontologies and annotations, protein–protein interactions, cis-regulatory information, protein information, gene expression data sets, sequence information and text-mining [26, 60]. We believed that the features from these databases could comprehensively measure the degree of similarity between AD candidate genes and known AD genes. Third, all the AD candidate genes are ranked based on their similarity scores with training genes, which results in one prioritization list for each data source. Finally, for fusing each of these rankings from the separate data sources into a single ranking and obtains a global prioritization, order statistics was used. A Q statistic was calculated for each candidate gene [60, 62]. The distribution of Q statistic was modeled by a gamma distribution, so we can get a *P*-value for every Q statistic. Based on this P-value, we can get a global ranking which combined rank of all separate ranks. In the global ranking, the genes rank forward means they are more similar with known AD genes, and on our hypothesis above, it is more likely to be an AD related gene. Here, the online software Endeavour (http://homes.esat.kuleuven.be/~bioiuser/endeavour/tool/endeavourweb.php) was used to realize this prioritization of candidate genes [26].

## SUPPLEMENTARY TABLES








